# Syndrome CHARGE avec tétralogie de Fallot: à propos d'un cas

**DOI:** 10.11604/pamj.2014.19.319.3543

**Published:** 2014-11-26

**Authors:** Fouad Amal Wahid, Aniss Seghrouchni, Abdedaim Elghadbane Hatim, Noureddine Atmani, Abdessamad Abdou, Abdelmajid Bouzerda, Sahar Mouram, Mohamed Drissi, Mahdi Ait Houssa, Abdelatif Boulahya

**Affiliations:** 1 Service de Chirurgie Cardiaque, Hôpital Militaire d'Instruction Mohammed V, Faculté de Médecine et de Pharmacie de Rabat, Université Mohammed V, Souissi, Rabat, Maroc; 2 Réanimation de Chirurgie Cardiaque, Hôpital Militaire d'instruction Mohammed V, Faculté de Médecine et de Pharmacie, Université Mohammed V, Souissi, Rabat, Maroc; 3 Service de Cardiologie, Hôpital Militaire d'Instruction Mohammed V, Faculté de Médecine et de Pharmacie, Université Mohammed V, Souissi, Rabat, Maroc

**Keywords:** Syndrome CHARGE, tétralogie de Fallot, colobome, atrésie des choanes, CHARGE Syndrome, tetralogy of Fallot, coloboma, choanal atresia

## Abstract

Le syndrome CHARGE est caractérisé par un large polymorphisme clinique associant colobome, anomalies cardiaques, atrésie de choanes, retard staturo-pondéral et de développement, anomalies génitales, anomalies des oreilles ainsi que d'autres anomalies. Les auteurs rapportent le cas d'un syndrome CHARGE diagnostiqué lors du bilan d'une tétralogie de Fallot chez un nourrisson de 22 mois. Les différentes manifestations cliniques de ce syndrome sont rapportées ainsi que les critères diagnostiques.

## Introduction

Le Syndrome de CHARGE est une entité clinique rare d'origine génétique associant à des degrés variables l'atteinte de multiples organes: colobome, anomalies cardiaques, atrésie des choanes, anomalies des oreilles ou surdité, anomalies génitales et un retard de développement psychomoteur et de croissance, nécessitant une approche multidisciplinaire pour sa prise en charge. Nous rapportons un cas de Syndrome CHARGE dont le diagnostic a été porté lors du bilan d'une tétralogie de Fallot chez un garçon de 22 mois.

## Patient et observation

**Figure 1:**
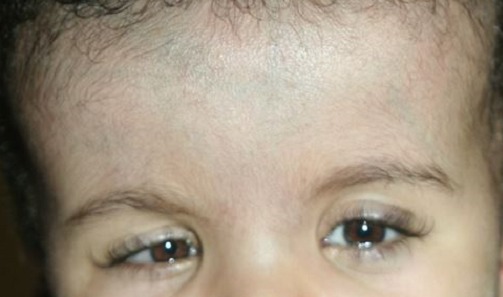
Dysmorphie faciale et microphtalmie

**Figure 2:**
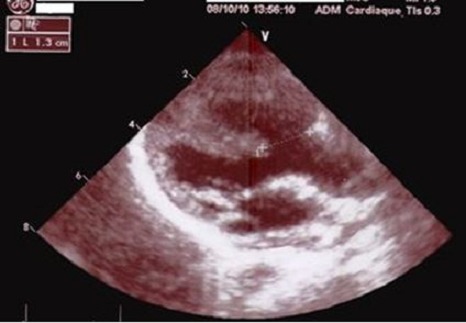
Échocardiographie montrant une tétralogie de FALLOT

**Figure 3:**
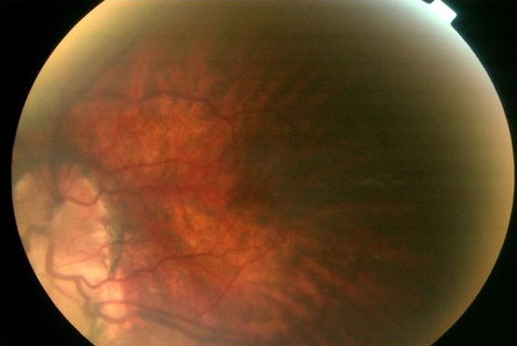
Colobome

Un nourrisson de sexe masculin âgé de 22 mois, né à domicile au terme d'une grossesse non suivie de parents sans antécédents particuliers, asymptomatique sur le plan cardiovasculaire se présente à la consultation de chirurgie cardiaque pour cardiopathie congénitale cyanogène découverte fortuitement lors de sa vaccination. Il présentait à l'examen physique un retard staturo-pondéral à 3 déviations standards (DS), une surdité, un retard des acquisitions psychomotrices avec une dysmorphie faciale par augmentation de la taille du crâne, une implantation basse des oreilles, un hypertélorisme et une microphtalmie bilatérale (Figure 1). L'examen cardiovasculaire a trouvé des signes cliniques, radiologiques, électriques et échocardiographiques d'une tétralogie de FALLOT avec sténose serrée de la voie pulmonaire (Figure 2). Le reste de l'examen somatique a montré une cryptorchidie et l'examen chez l'ophtalmologiste a trouvé un colobome (Figure 3). Le patient a été mis sous beta bloquant et supplémentation en fer en attendant la correction chirurgicale de sa cardiopathie. Il sera, en outre, confié pour un suivi neurologique et urologique.

## Discussion

Le syndrome CHARGE (OMIM 214800) a été décrit pour la première fois en 1979 simultanément par HILL et HUNT [1] ce qui lui valut la dénomination de syndrome de HILL et HUNT mais en 1981 R. PAGON lui donna le nom de syndrome CHARGE en utilisant l'acronyme des initiales des principales atteintes [2]; à savoir, colobome (Coloboma), anomalies cardiaques (Heart defect), atrésie des choanes (Choane Atresia), retard de croissance et de developpement psychomoteur (Retarded growth and development), hypoplasie génitale (Genital hypoplasia) et anomalies des oreilles et/ou surdité (Ear anomalies or deafness).

Il touche environ 1 personne sur 8500 à 10 000 naissances [3] ce qui en fait l'un des 25 syndromes malformatifs les plus fréquents [4] avec une distribution panéthnique sans distinction de fréquence pour les sexes. Il est le plus souvent dû à des mutations dans le gène CHD7 (chromodomain helicase DNA-binding protein-7 gene) [5]. La majorité des cas sont sporadiques et de rares cas familiaux à transmission autosomique dominante ont été rapportés [6].

**Manifestations cliniques:** le syndrome CHARGE se caractérise par un polymorphisme clinique associant à des degrés variables:

**Colobome:** c'est la principale anomalie, présente dans 85% des cas. I1 est le plus souvent bilateral [7]. I1 s'agit d'un défaut de fermeture de la fente colobomique lors de l'organogenèse. I1 peut atteindre plusieurs structures de l’œil. II est plus fréquemment, choriorétinien pur, mais il peut également toucher la papille optique et l'iris. Il s'accompagne d'une microphtalmie dans 40% des cas. Celle-ci est considérée comme un équivalent de colobome lorsqu'elle est isolée. Le pronostic visuel est essentiellement lié à la topographie papillaire éventuelle du colobome, à la bilatéralité des lésions et à la présence d'une microphtalmie [8].

**Anomalies cardiaques:** présentes dans 70 à 80% des cas. L'anomalie la plus fréquente est la tétralogie de Fallot mais d'autres anomalies peuvent exister comme la persistance du canal artériel, défauts septaux isolés et les sont les cardiopathies conotroncales [8].

**Atrésie des choanes:** présente dans plus de 40% des cas; elle est plus fréquemment osseuse que membraneuse et dans la moitié des cas bilaterale responsable dans la période néonatale de détresse respiratoire [8]. Sa présence indique un mauvais pronostic pour la survie et nécessite de multiples interventions chirurgicales complexes pour correction.

**Retard de croissance et de développement psychomoteur:** un retard de croissance est observé chez environ 75% des patients il peut être intra utérin dans un tiers des cas. Il est dû à des causes endocriniennes (par exemple un déficit en hormone de croissance ou en gonadotrophine) ou à des difficultés à s'alimenter. Le retard psychomoteur est conditionné par l'existence d'une malformation cérébrale, l'importance des handicaps neurosensoriels (qui doivent être détectés) et la précocité de leur prise en charge, ainsi que par le traitement rapide des problèmes chirurgicaux et la qualité du support familial [7]. Les patients présentant un colobome et des problèmes graves de l'oreille interne sont plus particulièrement touchés.

**Hypoplasie génitale:** i1 s'agit le plus souvent d'un micropénis et d'une cryptorchidie chez le garçon (80% des cas), et d'une hypoplasie des petites lèvres chez la fille (16%). Le retard de développement des organes génitaux externes est plus facile à reconnaitre chez les garçons que chez les filles, les principales anomalies sont un microphallus, agénésie du pénis, cryptorchidie, scrotum bifide, atrésie du vagin, hypoplasie des lèvres ou du clitoris.

**Anomalies des oreilles et surdité:** les trois parties de l'oreille peuvent être touchées. L'oreille externe est souvent petite, étroite et bas implantée (anomalies du pavillon, atrésie du conduit auditif, appendices pré-auriculaires). Le déficit sensoriel peut entrainer un audiogramme typique en triangle.

**Autres anomalies:** à coté de ces atteintes à l'origine de la dénomination du syndrome CHARGE, de nombreuses autres anomalies congénitales peuvent exister comme la paralysie faciale, des troubles du système nerveux central, des anomalies de déglutition, une fente labiale et / ou palatine, des malformations des voies urinaires ou fistule trachéo-œsophagienne.

**Diagnostic:** vu l'atteinte multifocale dans le syndrome charge, plusieurs auteurs ont proposé des critères diagnostiques se basant sur l'association de critères majeurs et de critères mineurs pour poser le diagnostic.

**Les Critères majeurs:** colobome, atrésie des choanes, anomalies des oreilles et anomalies des nefs crâniens.

**Les Critères mineurs:** atteinte cardiaque, les anomalies génitales, fente palatine, fente labiale, dysmorphie faciale, retard de croissance et retard de développement Initialement PAGON et al avaient proposé la présence de 4 critères pour poser le diagnostic avec présence obligatoire de colobome ou d'atrésie des choanes, d'autres auteurs ont proposé d'autres critères en fonction des analyses statistiques de leurs séries. Enfin, devant la multiplicité des formes cliniques Verloes a proposé la notion de syndrome CHARGE typique, atypique ou partiel.

Syndrome CHARGE typique: 3 critères majeurs ou 2 critères majeurs et 2 critères mineurs. Syndrome CHARGE partiel: 2 critères majeurs et 1 critère mineur; syndrome CHARGE atypique: 2 critères majeurs et o critère mineur. 1 critère majeur et 3 critères mineurs. Le pronostic du syndrome CHARGE est grevé par La coexistence de la cardiopathie avec une atrésie des choanes ou une fistule trachdo-oesophagienne [8].

A noter que le syndrome CHARGE peut être associé à d'autres syndromes comme le syndrome de Di George, le syndrome de Kallmann. Le diagnostic a été posé chez notre patient devant la présence de la dysmorphie faciale, surdité, colobome, microphtalmie, cryptorchidie, retard staturo-pondéral et de développement psychomoteur, tétralogie de Fallot. Soit un tableau correspondant à une forme typique selon VERLOES [9].

## Conclusion

Le syndrome CHARGE mérite d’être mieux connu car il pose le problème de diagnostic devant les critères mineurs et celui de prise en charge dans les formes sévères. Nous insistons sur la notion de prise en charge multidisciplinaire dans ce genre de maladie.
